# Vision impairment and food insecurity in the national health and aging trends study

**DOI:** 10.3389/fepid.2024.1353083

**Published:** 2024-05-01

**Authors:** Moon J. Lee, Louay Almidani, Laura Samuel, Bonnielin K. Swenor, Joshua R. Ehrlich, Varshini Varadaraj

**Affiliations:** ^1^ Wilmer Eye Institute, Johns Hopkins University School of Medicine, Baltimore, MD, United States; ^2^ Johns Hopkins Disability Health Research Center, Johns Hopkins University, Baltimore, MD, United States; ^3^ Johns Hopkins University School of Nursing, Baltimore, MD, United States; ^4^ Department of Ophthalmology and Visual Sciences, University of Michigan, Ann Arbor, MI, United States; ^5^ Institute for Social Research, University of Michigan, Ann Arbor, MI, United States

**Keywords:** vision impairment, food insecurity, aging, epidemiology, older adults

## Abstract

**Introduction:**

Vision impairment (VI) may further exacerbate older adults’ vulnerability to experiencing food insecurity and may be a unique and important target for policies addressing access to nutritional food. The purpose of this study is to determine the association of VI in older adults with food insecurity.

**Methods:**

This is a cross-sectional analysis of round 11 (2021) of the National Health and Aging Trends Study (NHATS), a nationally representative survey of U.S. Medicare beneficiaries ages 65 and older. Participants include 2,815 older adults with complete data on at least one objective measure of vision (distance, near or contrast sensitivity) and food insecurity. Food insecurity was assessed using a previously developed indicator of food insecurity in NHATS. VI was defined as binocular visual acuity (VA) worse than 0.3 logMAR (Snellen equivalent 20/40) at distance or its near equivalent, or contrast sensitivity (CS) worse than 1.55 logUnits. Continuous VI measures included distance and near VA (per 0.1 logMAR), and CS (per 0.1 logCS).

**Results:**

Participants were majority White (82%) and female (55%), and 3% had food insecurity. Older adults with any VI had a greater prevalence of food insecurity than adults without VI (5.0% vs. 2.0%, *p* < 0.05). In fully adjusted regression analyses, individuals with any VI experienced double the odds of food insecurity than individuals without VI (OR: 2.1, 95% CI: 1.2–3.6). Distance VI (measured continuously) was associated with 1.2 times the odds of food insecurity (OR = 1.2; 95% CI: 1.0–1.3, per 0.1 logMAR). All other vision measures trended towards higher odds of food insecurity, though not statistically significant.

**Discussion:**

Older adults with VI experience higher rates of food insecurity than their peers. Interventions to improve food security should be targeted towards addressing the specific barriers faced by visually impaired older adults.

## Introduction

Adults ages 65 and older comprise a rapidly growing proportion of the United States (U.S.) population with a projected increase by almost 18 million between the years 2020 and 2030 (1). Due to a multitude of individual and system level factors, older adults are at high risk of food insecurity, defined by the United States Department of Agriculture (USDA) as “limited or uncertain availability of nutritionally adequate and safe foods” (2, 3). Not only do older adults face specific social and financial constraints not faced by young populations such as higher rates of social isolation and reliance on fixed income sources, but they are also disproportionately impacted by age-related physical limitations, disabilities, and health conditions that impact their access to nutritional food (4, 5).

Vision impairment (VI) is also highly prevalent in the older adult population, with global estimates of 77% of 43.3 million blind individuals being age 50 and older (6). The presence of VI in older adults poses significant implications in terms of mobility (7), frailty (8, 9), and access to medical care and transportation (10), which can further magnify food insecurity.

Data on the association of VI and food insecurity is limited but evolving. Recent studies have demonstrated that both self-reported (trouble seeing) and objective measures of VI [presenting distance visual acuity (VA)] are associated with a greater prevalence of food insecurity in a dose-response manner (11, 12). However, there has been little data to date focusing on older adults and on other types of VI as measured by near VA, or contrast sensitivity (CS), in association with food insecurity, which may further help characterize this relationship. This is critical to identifying older adults with varying levels and types of VI that may be at increased vulnerability to experiencing food insecurity and developing targeted interventions.

Current interventions such as federal food assistance programs are in place, however, there are data gaps on the use of such programs by older adults with VI. The Supplemental Nutrition Assistance Program (SNAP) is currently the largest federally funded food assistance program in the U.S that provides monthly benefits to low-income individuals and families. In the fiscal year 2022, more than 41 million Americans per month participated in SNAP (13), and studies have shown that SNAP participation reduces food insecurity (14, 15). Therefore, we investigated the cross-sectional association between multiple objective measures of VI and food insecurity, and VI and SNAP participation, in a nationally representative survey of older adults.

## Materials and methods

### Survey data

We analyzed data from round 11 (2021) of the National Health and Aging Trends Study (NHATS), a nationally representative survey of U.S Medicare beneficiaries ages 65 and older. A total of 3,817 older adults completed in-person surveys alone or with the assistance of a proxy respondent. The primary analysis assessing the association between VI and food insecurity included a total of 2,815 community-dwelling participants after excluding adults who were deceased or nursing home residents, adults who did not attempt vision activities, and adults missing vision or food insecurity data (Supplementary Figure S1). The secondary analysis assessing the association between VI and SNAP status included 1,245 adults after excluding adults missing SNAP participation data, adults missing eligibility data and adults ineligible for SNAP participation (>200% of the federal poverty level) (16).

### Vision assessment

Binocular presenting VA was measured with participants wearing their usual corrective lenses and analyzed on categorical and continuous scales. All objective vision measures in NHATS were assessed using Ridgevue's tablet-based tests designed to replicate standard vision tests used in in-office eye examinations. This protocol was piloted in NHATS in 2019 and was found to have high levels of correlation when compared to the gold-standard equivalents for measuring VA and CS (17, 18). Distance and near VI was defined categorically as VA >0.3 logMAR (i.e., Snellen equivalent of <20/40) or its equivalent at near, respectively (19). Contrast sensitivity impairment (CSI) was defined as <1.55 logUnits, a cut off based on a prior study of normal CS values representing 2 standard deviations below the sample mean (18, 20). Any VI included participants who met VI criteria using distance VA, near VA, or CS. Continuous measures of distance and near VA, and CS were measured using a logMAR or logUnits scale, respectively. Detailed NHATS vision activities protocols have been described elsewhere (20).

### Other measurements

Food insecurity was defined using a summary indicator previously established using NHATS survey responses that was based on skipping meals, going without groceries, and going without hot meals due to lack of social support and/or financial barriers (21). In total, responses to 5 questions were assessed, and individuals with positive responses (“yes”) to any of the 5 items were classified as food insecure. The 5 questions included skipping meals due to lack of food or money to buy food, frequency of skipping meals (positive response defined as ≥ a few days), forgoing groceries due to lack of assistance, going without hot meals due to lack of assistance, and going without eating due to lack of assistance. SNAP participation status was assessed in all survey respondents based on the response “yes” to the question “There are several state and federal programs that help people in need. In the last year, did you receive help from SNAP?”, and was defined as SNAP eligible participants and SNAP eligible non-participants. Only SNAP eligible participants based on income were included in our secondary analysis.

Information on age (70–74, 75–79, 80–84, 85–89, ≥90 years), sex (male, female), race/ethnicity (Non-Hispanic White, Non-Hispanic African American, Hispanic, Non-Hispanic other), income (<22K, 22–40K, 40–75K, >75K), marital status Non-married/Non-partnered, and married/living with a partner), number of children in the household (0, ≥1) and comorbidities was also collected. Co-morbidities were categorized into total number of conditions (0–1, 2, 3, ≥4) which included: diabetes, hypertension, hyperlipidemia, arthritis, osteoarthritis, stroke, lung disease, cancer, and hip fractures.

### Statistical methods

All estimates incorporated survey weights to account for the complex survey design of NHATS and non-response. Sociodemographic characteristics were stratified by VI status and compared using Chi-squared tests for categorical variables and *t*-tests for continuous variables. Multivariable logistic regression models were used to assess the association between VI (any objective VI, distance VI, near VI, CSI) and food insecurity status. Models were adjusted for age, sex, race/ethnicity, income, marital status, number of children, and comorbidities. Covariates in the final model were included if demonstrated to be associated with VI and food insecurity and/or deemed clinically relevant. Any survey question responses of “Don't know” and “Refuse” were treated as missing values and excluded from analyses. For our secondary analysis, multivariable logistic regression models adjusting for the same confounders as the primary analysis, were used to investigate the association between VI and SNAP participation status.

In all analyses, income and education were found to be colinear, thus income alone was included in the primary model as it is a strong predictor of food insecurity and SNAP participation. However, for completeness, sensitivity analyses were conducted by adjusting for education in addition to the aforementioned covariates. In addition, sensitivity analyses were also conducted excluding marital status and number of children in the household as covariates due to the potential for conflation of these variables with food insecurity. Statistical significance was defined as *p* < 0.05. All analyses were conducted in Stata/SE 16.1 (StatCorp LLC, College Station, TX, USA).

## Results

### Sample characteristics

Of 2,815 community-dwelling NHATS participants, 1,075 individuals (unweighted *n*, weighted 32%) had any VI. Among these participants, 10% had distance VI, 21% had near VI, and 21% had CSI (non-exclusive groups). There were no individuals classified as blind. Compared to individuals without any VI, individuals with any VI were older (16% vs. 8% age 85–89, 9% vs. 3% age ≥90), had a higher proportion of Non-Hispanic White participants (26% vs. 15%), had lower income levels (30% vs. 14% with <22K), were more likely to be Non-married/Non-partnered (59% vs. 45%), and had more children (23% vs. 15% with ≥1 child) (*p* < 0.05 for all) (Table 1).

**Table 1:** Participants’ characteristics by visual impairment status, NHATS 2021.

	Total Study Sample	Any objective VI	No objective VI	*P*-value	Distance VI (<20/40)	No Distance VI (≥ 20/40)	*P*-value	Near VI (<20/40)	No Near VI (≥ 20/40)	*P*-value	CSI (<1.55 LogCS)	No CSI (≥1.55 LogCS)	*P*-value
Participants, *n* (%)	2,815	1,075 (32)	1,740 (68)		364 (10)	2,451 (90)		719 (21)	2,096 (79)		702 (21)	2,113 (79)	
Age Groups, *n* (%)													
70–74	411 (31)	107 (22)	304 (34)	<.001	25 (14)	386 (32)	<.001	69 (22)	342 (33	<.001	54 (19)	357 (33)	<.001
75–79	881 (34)	259 (30)	622 (36)		79 (30)	802 (34)		177 (29)	704 (35)		154 (30)	727 (35)	
80–84	725 (20)	275 (22)	450 (19)		85 (23)	640 (20)		178 (22)	547 (19)		180 (22)	545 (19)	
85–89	495 (11)	245 (16)	250 (8)		85 (18)	410 (10)		163 (16)	332 (9)		181 (19)	314 (9)	
≥90	303 (5)	189 (9)	114 (3)		90 (15)	213 (4)		132 (10)	171 (4)		133 (10)	170 (4)	
Food security, *n* (%)													
Food insecure	98 (3)	56 (5)	42 (2)	<.001	21 (5)	77 (2)	.02	38 (5)	60 (2)	.008	40 (4)	58 (2)	.03
Gender, *n* (%)													
Female	1,603 (55)	604 (54)	999 (55)	.88	204 (54)	1,399 (55)	.76	405 (55)	1,198 (55)	.75	401 (56)	1,202 (55)	.68
Race/Ethnicity, *n* (%)													
White	2,012 (82)	680 (74)	1,332 (85)	<.001	225 (74)	1,787 (82)	.03	446 (74)	1,566 (84)	<.001	445 (74)	1,567 (84)	.001
African American	575 (8)	290 (12)	285 (6)		110 (14)	465 (7)		200 (12)	375 (7)		184 (11)	391 (7)	
Hispanic	132 (7)	66 (10)	66 (6)		21 (9)	111 (7)		45 (10)	87 (6)		46 (10)	86 (6)	
Other	63 (3)	26 (4)	37 (3)		6 (4)	57 (3)		17 (4)	46 (3)		18 (5)	45 (3)	
Education, *n* (%)													
≤High school	1,148 (36)	522 (43)	626 (32)	<.001	195 (47)	953 (35)	.003	364 (45)	784 (34)	<.001	338 (44)	810 (34)	.003
Some college/Vocational	610 (23)	212 (21)	398 (24)		69 (20)	541 (23)		148 (21)	462 (23)		132 (21)	478 (23)	
≥College graduate	1,049 (41)	335 (36)	714 (44)		98 (33)	951 (42)		202 (34)	847 (43)		227 (35)	822 (43)	
Total income (USD), *n* (%)													
<22K	673 (19)	363 (30)	310 (14)	<.001	133 (31)	540 (18)	<.001	249 (30)	424 (16)	<.001	254 (32)	419 (16)	<.001
22–40K	652 (21)	271 (24)	381 (20)		94 (21)	558 (21)		183 (25)	469 (20)		170 (24)	482 (20)	
40–75K	736 (28)	245 (22)	491 (31)		76 (24)	660 (29)		162 (23)	574 (30)		152 (21)	584 (30)	
>75K	754 (32)	196 (24)	558 (35)		61 (24)	693 (32)		125 (22)	629 (34)		126 (23)	628 (34)	
Marital status, *n* (%)													
Married/Living with partner	1,324 (55)	406 (45)	918 (59)	<.001	121 (40)	1,203 (56)	<.001	259 (42)	1,065 (58)	<.001	255 (44)	1,069 (58)	<.001
Separated/Divorced/Widowed/Never married	1,491 (45)	669 (55)	822 (41)		243 (60)	1,248 (44)		460 (58)	1,031 (42)		447 (56)	1,044 (42)	
Number of children, *n* (%)													
0	2,236 (83)	790 (77)	1,446 (85)	.001	256 (72)	1,980 (84)	<.001	521 (77)	1,715 (84)	<.001	507 (77)	1,729 (84)	.001
≥1	579 (17)	285 (23)	294 (15)		108 (28)	471 (16)		198 (23)	381 (16)		195 (23)	384 (16)	
Number of comorbidities, *n* (%)													
0–1	497 (20)	175 (18)	322 (21)	.004	55 (16)	442 (21)	.11	112 (17)	385 (21)	.001	100 (15)	397 (22)	.002
2	728 (28)	260 (26)	468 (29)		81 (24)	647 (29)		168 (25)	560 (29)		160 (26)	568 (29)	
3	767 (26)	288 (24)	479 (26)		113 (29)	654 (25)		192 (25)	575 (26)		195 (25)	572 (26)	
≥4	823 (26)	352 (31)	471 (23)		115 (31)	708 (25)		247 (33)	576 (24)		247 (34)	576 (24)	
Visual functioning measures													
Distance VA (logMAR), median (IQR)	0.10 (0, 0.20)	0.22 (0.12, 0.34)	0.04 (−0.04, 0.12)	<.001	0.44 (0.36, 0.56)	0.08 (−0.02, 0.16)	<.001	0.24 (0.14, 0.38)	0.06 (−0.02, 0.16)	<.001	0.24 (0.12, 0.38)	0.06 (−0.02, 0.16)	<.001
Near VA (logMAR), median (IQR)	0.18 (0.09, 0.28)	0.35 (0.24, 0.45)	0.13 (0.06, 0.20)	<.001	0.41 (0.27, 0.62)	0.16 (0.08, 0.25)	<.001	0.40 (0.35, 0.52)	0.14 (0.07, 0.21)	<.001	0.32 (0.20, 0.47)	0.15 (0.07, 0.23)	<.001
CS (logCS), median (IQR)	1.80 (1.60, 1.85)	1.50 (1.35, 1.65)	1.80 (1.75, 1.85)	<.001	1.40 (1.15, 1.60)	1.80 (1.65, 1.85)	<.001	1.50 (1.35, 1.70)	1.80 (1.65, 1.85)	<.001	1.40 (1.25, 1.45)	1.80 (1.70, 1.85)	<.001

VA, visual acuity; CS, contrast sensitivity; VI, vision impairment; CSI, contrast sensitivity impairment; logMAR, logarithm of the minimum angle of resolution. *N* is unweighted; % is survey weighted estimates.

### Prevalence of food insecurity by VI status

**Figure 1:**
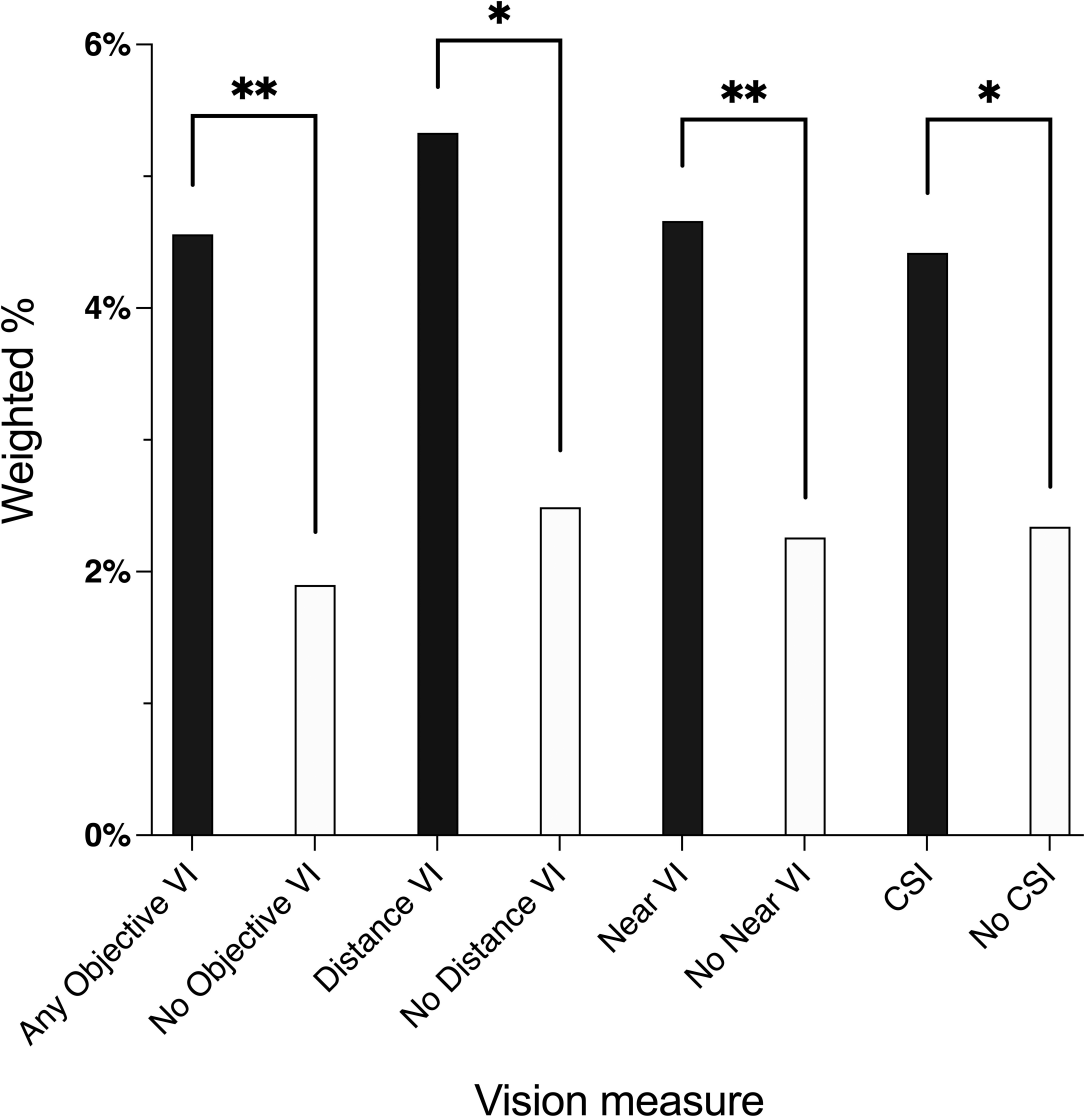
Weighted prevalence of food insecurity by vision impairment: national health and aging trends study, 2021. VI, vision impairment; CSI, contrast sensitivity impairment. *p < 0.05, **p < 0.01.

The overall prevalence of community-dwelling older adults with food insecurity was low (3%, weighted). There was a higher proportion of older adults with food insecurity in every VI group as compared to the respective non-visually impaired groups (any VI vs. no VI: 5% vs. 2%; distance VI vs. no distance VI: 5% vs. 2%; near VI vs. no near VI: 5% vs. 2%, CSI vs. no CSI: 4% vs. 2%; all *p* < 0.05, Figure 1).

### Primary regression analyses: food insecurity

In multivariable regression analyses, as compared to older adults without any VI, adults with any VI were more likely to have food insecurity (OR = 2.1; 95% CI: 1.2–3.6), Table 2. Older adults with distance VI, near VI, and CSI as measured categorically, trended towards being more likely to have food insecurity than their respective non-visually impaired counterparts, though this was not statistically significant (distance VI vs. no distance VI: OR = 1.8; 95% CI: 0.9–3.8; near VI vs. no near VI: OR = 1.7; 95% CI: 0.9–2.9; CSI vs. no CSI: OR = 1.5; 95% CI: 0.8–2.9).

**Table 2:** Associations of vision impairment with food insecurity status in NHATS 2021^a^.

Associations of vision measures with food insecurity, national health and aging trends study 2021			
VI Type	Interval	OR (95% CI)	*P*-value
Any objective VI	No objective VI	**2.08** (**1.20–3.58)**	.009
Distance VI			
Categorical	No distance VI	1.81 (0.88–3.76)	.11
Continuous	0.1 logMAR worse	**1.15** (**1.04–1.27)**	.008
Near VI			
Categorical	No near VI	1.65 (0.92–2.94)	.08
Continuous	0.1 logMAR worse	1.09 (1.00–1.19)	.06
CS impairment			
Categorical	No CSI	1.52 (0.81–2.87)	.19
Continuous	0.1 logCS worse	1.08 (0.99–1.18)	.08

OR, Odds ratio for food insecure vs. food secure in logistic regression; CI, confidence interval; VI, vision impairment; CS, contrast sensitivity.

^a^ All models accounted for survey design and were adjusted for age, sex, race/ethnicity, income, marital status, number of children, and comorbidities.

The bolded values are the statistically significant values with *p* < 0.05.

Using continuous measures of VA, those with worse distance VA were more likely to have food insecurity (OR = 1.2 for every 0.1 logMAR decrement in distance VA; 95% CI: 1.0–1.43). Similarly, continuous measures of near VA and CS also trended towards higher likelihood of food insecurity with decrements in near VA and CS, though this was not statistically significant (OR = 1.1 for every 0.1 logMAR decrement in near VA; 95% CI: 1.0–1.2), (OR = 1.1 for every 0.1 logCS decrement in CS; 95% CI: 1.0–1.2).

In sensitivity analysis with education included as an additional covariate, the results were similar. Final models shown included income alone for statistical parsimony. Additional sensitivity analysis excluding marital status and number of children as covariates also showed that older adults with any VI had a higher odds of food insecurity (OR = 2.0; 95% CI: 1.2–3.4) than adults without VI. Older adults with worse distance VA measured continuously also had a higher odds of food insecurity (OR = 1.2; 95% CI: 1.1–1.3, per 0.1 logMAR decrement in VA).

### Prevalence of SNAP participation by VI status

**Figure 2:**
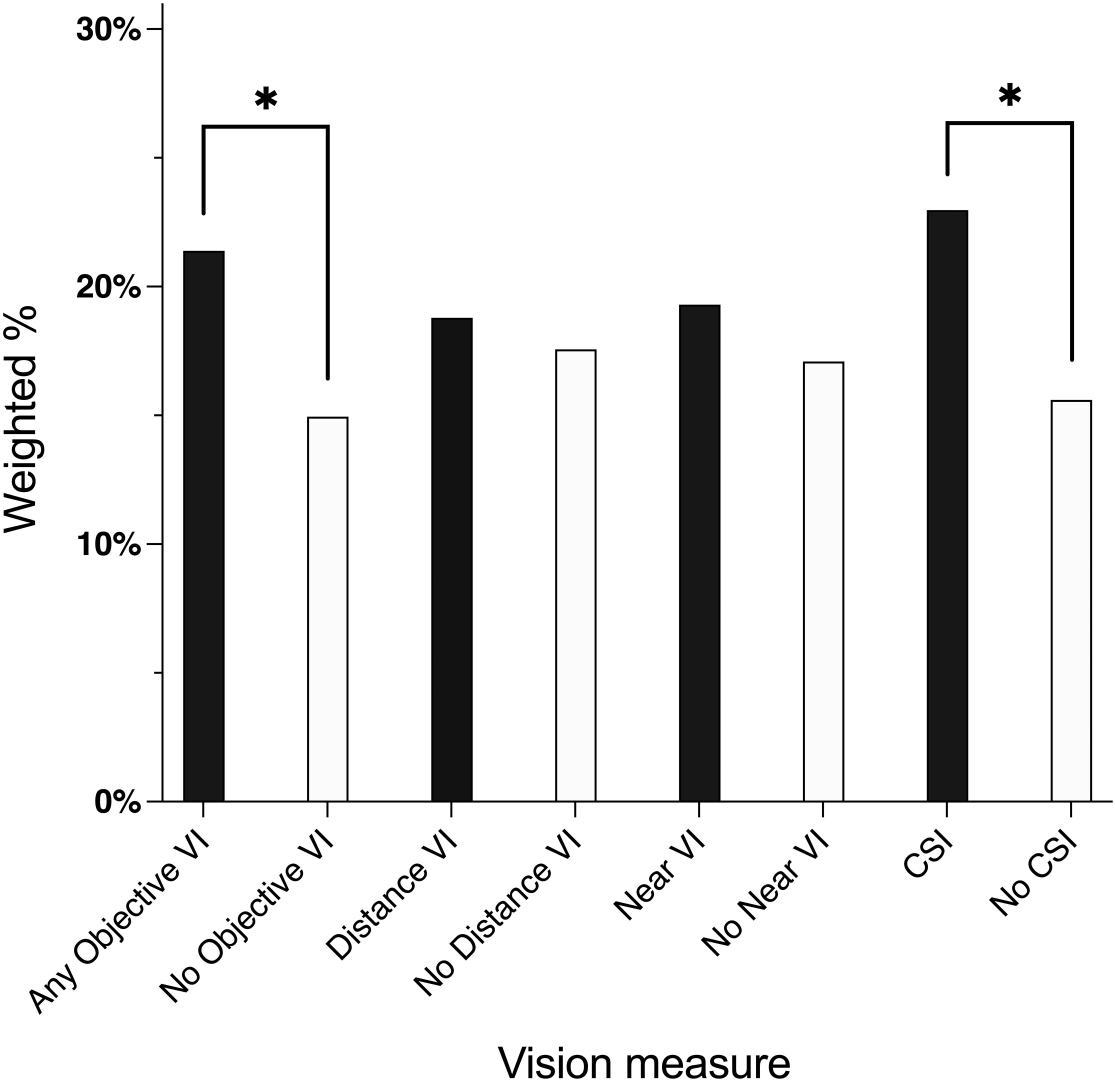
Weighted prevalence of SNAP participation by vision impairment: national health and aging trends study, 2021. VI, vision impairment; CSI, contrast sensitivity impairment. * *p* < 0.05, ** *p* < 0.01.

There was a higher proportion of older adults who were SNAP participants among those with any VI than adults without any VI (21.4% vs. 15% respectively, *p* < 0.05), Figure 2. There was no difference in SNAP participation status by distance or near VI status. There was also a higher proportion of SNAP participation in older adults with CSI compared to no CSI (22.9% vs. 16.6% respectively, *p* < 0.05).

### Secondary regression analyses: SNAP participation

In a secondary multivariable regression analysis, older adults with any VI as compared to adults without any VI trended towards a higher odds of SNAP participation, however this was not statistically significant (OR = 1.5; 95% CI: 1.0–2.2) (Table 3). Categorical and continuous measures of both distance and near VI were not associated with SNAP participation status. Older adults with CSI had a higher-odds of participating in SNAP (OR = 1.6, 95% CI: 1.0–2.4) when measured categorically. On a continuous scale, for every decrement of 0.1 logUnits in CS, there was a trend towards higher-odds of participating in SNAP though not statistically significant (OR = 1.1; 95% CI 1.0–1.2).

**Table 3:** Associations of vision impairment with SNAP participant status in NHATS 2021^a^.

Associations of vision measures with SNAP participant status, national health and aging trends study 2021			
Vision impairment type	Interval	OR (95% CI)	*P*-value
Any objective VI	No objective VI	1.47 (0.98–2.21)	.06
Distance VI			
Categorical	No distance VI	1.17 (0.61–2.24)	.64
Continuous	0.1 logMAR worse	1.09 (0.99–1.20)	.09
Near VI			
Categorical	No near VI	1.09 (0.73–1.63)	.66
Continuous	0.1 logMAR worse	1.03 (0.94–1.13)	.49
CSI			
Categorical	No CSI	**1.57** (**1.01–2.43)**	.04
Continuous	0.1 logCS worse	1.08 (1.00–1.16)	.06

SNAP, supplemental nutrition assistance program; OR, Odds ratio for SNAP participation vs. no SNAP participation in logistic regression; CI, confidence interval; VI, vision impairment; CSI, contrast sensitivity impairment.

^a^ All models accounted for survey design and were adjusted for age, sex, race/ethnicity, income, marital status, number of children, and comorbidities.

The bolded values are the statistically significant values with *p* < 0.05.

## Discussion

In this cross-sectional, nationally representative survey of community-dwelling older adults with Medicare, participants with VI were more likely to experience food insecurity than their non-visually impaired counterparts. There was no difference in SNAP participation status in older adults with VI compared to adults without. Older adults with any VI (distance VI, near VI, or CSI) had twice the odds of food insecurity compared to adults without objective VI. While individual categorical measures of VI (distance VI, near VI, and CSI) were not significantly associated with food insecurity, distance VI measured continuously was associated with a 1.2-fold higher odds of food insecurity. Overall, these results suggest that older adults with objective VI may be at an increased risk of experiencing food insecurity, and that this relationship may differ based on the type of VI.

While prior studies have demonstrated associations between VI and food insecurity, there has been little data on the relationship between different types of objective VI and food insecurity among older adults. Elucidating these relationships is important as the type of VI may have differing implications for daily function and therefore food access. For example, numerous studies have shown the differential effects of DVI, NVI and CSI on functioning and frailty (a syndrome of aging-related physiological decline characterized by increased vulnerability to stressors and adverse health outcomes) (8, 9, 22, 23). Of these vision measures, some studies have suggested that DVI may potentially have a stronger association with frailty (24). Ultimately, specific types of targeted interventions may be required based on type of VI to improve food insecurity.

Our results are in agreement with a prior study of visually impaired U.S adults ages 50 and older which found that food insecurity was associated with both objective presenting VI and subjective VI in a dose-response manner (11). Kolli et al. also found that associations between food insecurity and VI were magnified in U.S adults 65 and older compared to those less than 65 years of age (11). Compared to our study, Kolli et al. had a much larger sample size and may have been powered to detect smaller differences, but only assessed distance VA and did not account for other measures of VI. Their study also assessed food insecurity differently from our study, as they used the full 10-item U.S. Household Food Security Survey Module. In another cross-sectional study of low-income U.S adults ≥18 years of age, also using the 10-item U.S. Household Food Security Survey Module, adults with subjective VI had a 216% higher odds of being food insecure as compared to adults without VI, but did not include objective vision measures (12).

This study found that the overall prevalence of food insecurity among U.S. older adults was low (3%, weighted). This is lower than estimates obtained in prior studies, with the U.S. Department of Agriculture Economic Research Service reporting 7.1% of older adults as food insecure in 2021 (25). However, our study used a different food insecurity measure, which asks about inability to obtain food rather than difficulty obtaining food (26). Therefore, the measure used in this study may capture the most severe form of food insufficiency and builds on prior findings by showing that those with VI are more likely than their peers to lack sufficient food.

The relationship between VI and food insecurity is complex and multifactorial. Individuals with VI have greater difficulty accessing food due to transportation barriers, lack of accessible grocery stores, difficulty preparing food, and affording nutritious food (27). Overarching disparities in employment, education and income also contribute to the financial strain of affording food as there are competing interests such as affording housing and medical care (28, 29, 30). In addition to VI contributing to food insecurity in older adults, a second bi-directional relationship likely also exists with food insecurity and poor nutrition contributing to the progression and development of various ophthalmic diseases (11, 12). For example, numerous studies have shown that household food insecurity was associated with lack of ophthalmic care, developing diabetic retinopathy (DR) and losing vision to DR (31, 32). More generally, unhealthy diets have been identified as a risk factor for the development of nuclear cataracts and early age-related macular degeneration (33, 34). In order to fully elucidate the complex bi-directional relationship between VI and food insecurity, longitudinal data are needed.

In assessing the association between VI and SNAP participation status, our study found no difference in odds of SNAP participation by VI status, despite older adults being more likely to be food insecure. Although this may be due to a relatively smaller sample size, it may also in part, be due to the challenges faced by individuals with disabilities in enrolling for SNAP. Prior work has shown that the online SNAP enrollment process differs by state and vary in their levels of flexibility, efficiency and accessibility, suggesting that people with disabilities may face barriers in enrollment based on geographic location (35). More specifically, a large proportion of state SNAP websites do not offer mail-in (25%) or telephone (75%) enrollment options, limiting the flexibility of enrollment methods for people with VI in particular (35). In addition, SNAP and various other food assistance programs have eligibility rules for individuals with disability based on receipt of disability benefits which may not incorporate the full spectrum of individuals with VI that are at increased risk of food insecurity (36). SNAP participation is important as it has been shown to effectively decrease food insecurity (37). It is also associated with decreased health care expenditures and improved health outcomes for diabetics (16, 38). Thus, improving SNAP participation rates in older adults with VI will be an important indicator of efforts to alleviate food insecurity in this particularly vulnerable population. In addition to improving accessibility of enrollment for programs such as SNAP, broader efforts focused on universal design are needed to allow individuals with VI equal access to food and food assistance programs. This will incorporate addressing the various barriers individuals with VI may face including transportation, accessibility of grocery stores, and preparing and affording nutritious food.

Strengths of this study include the use of a nationally representative sample, objective measures of VI, and multiple visual function measures including distance and near acuity, and CS. To the best of our knowledge, there are few studies that have examined the association between various measures of objective VI and SNAP participation status among Medicare beneficiaries.

This study has some limitations. First, our analyses were limited by sample size, making it difficult to distinguish whether true differences exist in the associations of different measures of objective VI and food insecurity or whether the analysis was not powered to detect these differences. In addition, a large number of survey respondents were excluded in the analysis due to missing data. Second, this was a cross-sectional analysis, and longitudinal data are needed to further elucidate the temporality and directionality of the association between VI and food insecurity. Third, the NHATS summary indicator used to assess food insecurity is based on survey responses to 5 questions which do not capture hunger or the degree of difficulty obtaining food. While this indicator of food insecurity has been validated in the NHATS population (21), it is shorter than the USDA food insecurity survey module which includes 10 questions and 8 additional questions for households with children and may capture a broader and different dimension of food insecurity. Fourth, while our analyses included number of comorbidities, we did not control for the presence of additional non-vision related disabilities, which may confound the relationship between VI and food insecurity, as well as participation in SNAP by visually impaired individuals.

In conclusion, our study found that VI, as measured by distance or near VA, or CS, was associated with food insecurity, but was not associated with participation in SNAP. Older adults and individuals with disabilities are more likely to experience food insecurity, and this may be further amplified in older adults with VI who experience difficulty with mobility and activities of daily living (39, 40, 41). Further longitudinal studies are needed to understand the likely bi-directional nature of this relationship and to help target solutions to improving food insecurity in older adults with VI.

## Data Availability

The original contributions presented in the study are included in the article/**Supplementary Materials**, further inquiries can be directed to the corresponding author. The NHATS dataset used in our manuscript is a publicly available dataset that is available here: https://nhats.org/researcher/nhats.

## References

[1] Mather M Jacobsen LA Pollard KM. Aging in the United States. Popul Bull. (2015) 70(2):375–90. 10.1016/B978-0-12-373932-2.00293-3

[2] Bailey LB Campbell CC Cohen BE Dewey KG Dietz WH Dwyer J Core indicators of nutritional state for difficult-to-sample populations. J Nutr. (1990) 120(11 SUPPL.):1555–98. 10.1093/JN/120.SUPPL_11.1555 2243305

[3] National Academies of Sciences, E. and M. Social Isolation and Loneliness in Older Adults: Opportunities for the Health Care System. Washington, DC: National Academies Press (US) (2020). 10.17226/25663 32510896

[4] Heflin CM Altman CE Rodriguez LL. Food insecurity and disability in the United States. Disabil Health J. (2019) 12(2):220–6. 10.1016/J.DHJO.2018.09.006 30322776

[5] Lee JS Fischer JG Johnson MA. Food insecurity, food and nutrition programs, and aging: experiences from Georgia. J Nutr Elder. (2010) 29(2):116–49. 10.1080/01639366.2010.480895 20473809

[6] Burton MJ Ramke J Marques AP Bourne RRA Congdon N Jones I The lancet global health commission on global eye health: vision beyond 2020. Lancet Glob Health. (2021) 9(4):e489–551. 10.1016/S2214-109X(20)30488-5/ATTACHMENT/81825AA4-162A-46FA-9EDD-70C02455686A/MMC3.PDF 33607016 PMC7966694

[7] Swenor BK Simonsick EM Ferrucci L Newman AB Rubin S Wilson V. Visual impairment and incident mobility limitations: the health, aging and body composition study. J Am Geriatr Soc. (2015) 63(1):46–54. 10.1111/JGS.13183 25536849 PMC4300238

[8] Swenor BK Lee MJ Tian J Varadaraj V Bandeen-Roche K. Visual impairment and frailty: examining an understudied relationship. J Gerontol A Biol Sci Med Sci. (2020) 75(3):596–602. 10.1093/gerona/glz182 31419280 PMC7328203

[9] Varadaraj V Lee MJ Tian J Ramulu PY Bandeen-Roche K Swenor BK. Near vision impairment and frailty: evidence of an association. Am J Ophthalmol. (2019) 208:234–41. 10.1016/J.AJO.2019.08.009 31465753 PMC6888870

[10] Spencer C Frick K Gower EW Kempen JH Wolff JL. Disparities in access to medical care for individuals with vision impairment. Ophthalmic Epidemiol. (2009) 16(5):281–8. 10.1080/09286580902999439 19874107

[11] Kolli A Mozaffarian R Kenney EL. Food insecurity and vision impairment among adults age 50 and older in the United States. Am J Ophthalmol. (2022) 236:69–78. 10.1016/j.ajo.2021.10.002 34653357

[12] Kumar P Brinson J Wang J Samuel L Swenor BK Scott AW Self-reported vision impairment and food insecurity in the US: national health interview survey, 2011–2018. Ophthalmic Epidemiol. (2022) 00(00):1–9. 10.1080/09286586.2022.2129698 PMC10987945 36204819

[13] USDA Food and Nutrition Service. Supplemental Nutrition Assistance Program Participation and Costs. (2023). p. 1–2.

[14] Mabli J Ohls J Dragoset L Castner L Santos B. Measuring the Effect of Supplemental Nutrition Assistance Program (SNAP) Participation on Food Security (2013). p. 1–356. Available online at: https://ageconsearch.umn.edu/record/339046/?v=pdf (Accessed February 15, 2024).

[15] Ratcliffe C McKernan SM Zhang S. How much does the supplemental nutrition assistance program reduce food insecurity? Am J Agric Econ. (2011) 93(4):1082–98. 10.1093/AJAE/AAR026 25197100 PMC4154696

[16] Berkowitz SA Seligman HK Rigdon J Meigs JB Basu S. Supplemental nutrition assistance program (SNAP) participation and health care expenditures among low-income adults. JAMA Intern Med. (2017) 177(11):1642–9. 10.1001/jamainternmed.2017.4841 28973507 PMC5710268

[17] Hu M Freedman VA Ehrlich JR Reed NS Billington C Kasper JD. Collecting objective measures of visual and auditory function in a national in-home survey of older adults. J Surv Stat Methodol. (2021) 9:309–34. 10.1093/jssam/smaa044 33869640 PMC8027590

[18] Varadaraj V Assi L Gajwani P Wahl M David J Swenor BK Ehrlich JR. Evaluation of tablet-based tests of visual acuity and contrast sensitivity in older adults. Ophthalmic Epidemiol. (2021) 28(4):293–300. 10.1080/09286586.2020.1846758 33185485 PMC8116354

[19] American Academy of Ophthalmology. US Eye Disease Statistics. (2017). Available online at: https://www.aao.org/eye-disease-statistics (Accessed March 1, 2024).

[20] Hu M Erlich JR Reed NS Freedman VA. National Health and Aging Trends Study (NHATS) Vision and Hearing Acitivites User Guide. 2. European University Institute (2022). p. 1–31. Available online at: https://eur-lex.europa.eu/legal-content/PT/TXT/PDF/?uri=CELEX:32016R0679&from=PT%0A, http://eur-lex.europa.eu/LexUriServ/LexUriServ.do?uri=CELEX:52012PC0011:pt:NOT

[21] Tucher EL Keeney T Cohen AJ Thomas KS. Conceptualizing food insecurity among older adults: development of a summary indicator in the national health and aging trends study. J Gerontol B Psychol Sci Soc Sci. (2021) 76(10):2063–72. 10.1093/geronb/gbaa147 33001172 PMC8599055

[22] Haymes SA Johnston AW Heyes AD. Relationship between vision impairment and ability to perform activities of daily living. Ophthalmic Physiol Opt. (2022) 22(2):79–91. 10.1046/j.1475-1313.2002.00016.x 12014491

[23] Owsley C Sloane ME. Contrast sensitivity, acuity, and the perception of “real-world” targets. Br J Ophthalmol. (1987) 71:791–6. 10.1136/bjo.71.10.791 3676151 PMC1041308

[24] Sonnenfeld ML Pappadis MR Reistetter TA Raji MA Ottenbacher K Al Snih S Vision impairment and frailty among Mexican American older adults: a longitudinal study. J Appl Gerontol. (2024):1–10. 10.1177/07334648241231374 PMC11052670 38412864

[25] Coleman-Jensen A Rabbitt MP Gregory CA Singh A. Household Food Security in the United States in 2021. (n.d.). Available online at: www.ers.usda.gov (retrieved May 14, 2023) 10.1016/j.jand.2023.03.012 37730304

[26] Bickel GW, United States, Food and Nutrition Service, Office of Analysis, Nutrition, and E. (2000). US Adult Food Security Survey Module. USDA Publications.

[27] Sahingoz SA. Visually impaired consumers and food shopping. Br J Human Soc Sci. (2012) 7:63–74.

[28] Besagar S Yonekawa Y Sridhar J Finn A Padovani-Claudio DA Sternberg P Association of socioeconomic, demographic, and health care access disparities with severe visual impairment in the US. JAMA Ophthalmol. (2022) 140(12):1219–26. 10.1001/JAMAOphthalmol.2022.4566 36326732 PMC9634598

[29] Lansingh V Carter M Ulldemolins A Valencia L Eckert K. Social inequalities in blindness and visual impairment: a review of social determinants. Indian J Ophthalmol. (2012) 60(5):368. 10.4103/0301-4738.100529 22944744 PMC3491260

[30] Mcdonnall MC Tatch A. Educational attainment and employment for individuals with visual impairments. J Visual Impair Blin. (2021) 115(2):152–9. 10.1177/0145482X211000963

[31] Gibson DM. Food insecurity, eye care receipt, and diabetic retinopathy among US adults with diabetes: implications for primary care. J Gen Intern Med. (2019) 34(9):1700–2. 10.1007/s11606-019-04992-x 30972552 PMC6712119

[32] Mosley-Johnson E Walker RJ Nagavally S Hawks L Bhandari S Trasser H Relationship between food insecurity and housing instability on quality of care and quality of life in adults with diabetes. PLoS One. (2022) 17(12):1–12. 10.1371/journal.pone.0278650 PMC9725149 36472986

[33] Mares JA Voland R Adler R Tinker L Millen AE Moeller SM Healthy diets and the subsequent prevalence of nuclear cataract in women. Arch Ophthalmol. (2010) 128(6):738–49. 10.1001/ARCHOPHTHALMOL.2010.84 20547952 PMC2896219

[34] Mares JA Voland RP Sondel SA Millen AE LaRowe T Moeller SM Healthy lifestyles related to subsequent prevalence of age-related macular degeneration. Arch Ophthalmol. (2011) 129(4):470–80. 10.1001/ARCHOPHTHALMOL.2010.314 21149749 PMC3075357

[35] Samuel LJ Xiao E Cerilli C Sweeney F Campanile J Milki N The development of the supplemental nutrition assistance program enrollment accessibility (SNAP-access) score. Disabil Health J. (2022) 15(4):101366. 10.1016/J.DHJO.2022.101366 36041996 PMC10987943

[36] Samuel LJ Zhu J Dwivedi P Stuart EA Szanton SL Li Q Food insecurity gaps in the supplemental nutrition assistance program based on disability status. Disabil Health J. (2023) 16(4):101486. 10.1016/J.DHJO.2023.101486 37353370 PMC10527001

[37] Mabli J Ohls J. Supplemental nutrition assistance program participation is associated with an increase in household food security in a national evaluation. J Nutr. (2015) 145(2):344–51. 10.3945/JN.114.198697 25644357

[38] Mayer VL McDonough K Seligman H Mitra N Long JA. Food insecurity, coping strategies and glucose control in low-income patients with diabetes. Public Health Nutr. (2016) 19(6):1103–11. 10.1017/S1368980015002323 26328922 PMC10270824

[39] Lam BL Christ SL Zheng DD West SK Munoz BE Swenor BK Longitudinal relationships among visual acuity and tasks of everyday life: the Salisbury eye evaluation study. Invest Ophthalmol Vis Sci. (2013) 54(1):193. 10.1167/iovs.12-10542 23221066 PMC3544421

[40] Swenor BK Muñoz B West SK. Does visual impairment affect mobility over time? The Salisbury eye evaluation study. Invest Ophthalmol Visual Sci. (2013) 54(12):7683–90. 10.1167/IOVS.13-12869 24176902 PMC3835273

[41] Swenor BK Muñoz B West SK. A longitudinal study of the association between visual impairment and mobility performance in older adults: the Salisbury eye evaluation study. Am J Epidemiol. (2014) 179(3):313–22. 10.1093/aje/kwt257 24148711 PMC3954103

