# Supplementary material for: Vision impairment and food insecurity in the national health and aging trends study
Source: Front Epidemiol. 2024 May 1;4:1353083. doi: 10.3389/fepid.2024.1353083 (PMC11094228; doi:10.3389/fepid.2024.1353083)
Supplement: Supplementary file 1 [file Datasheet1.docx]

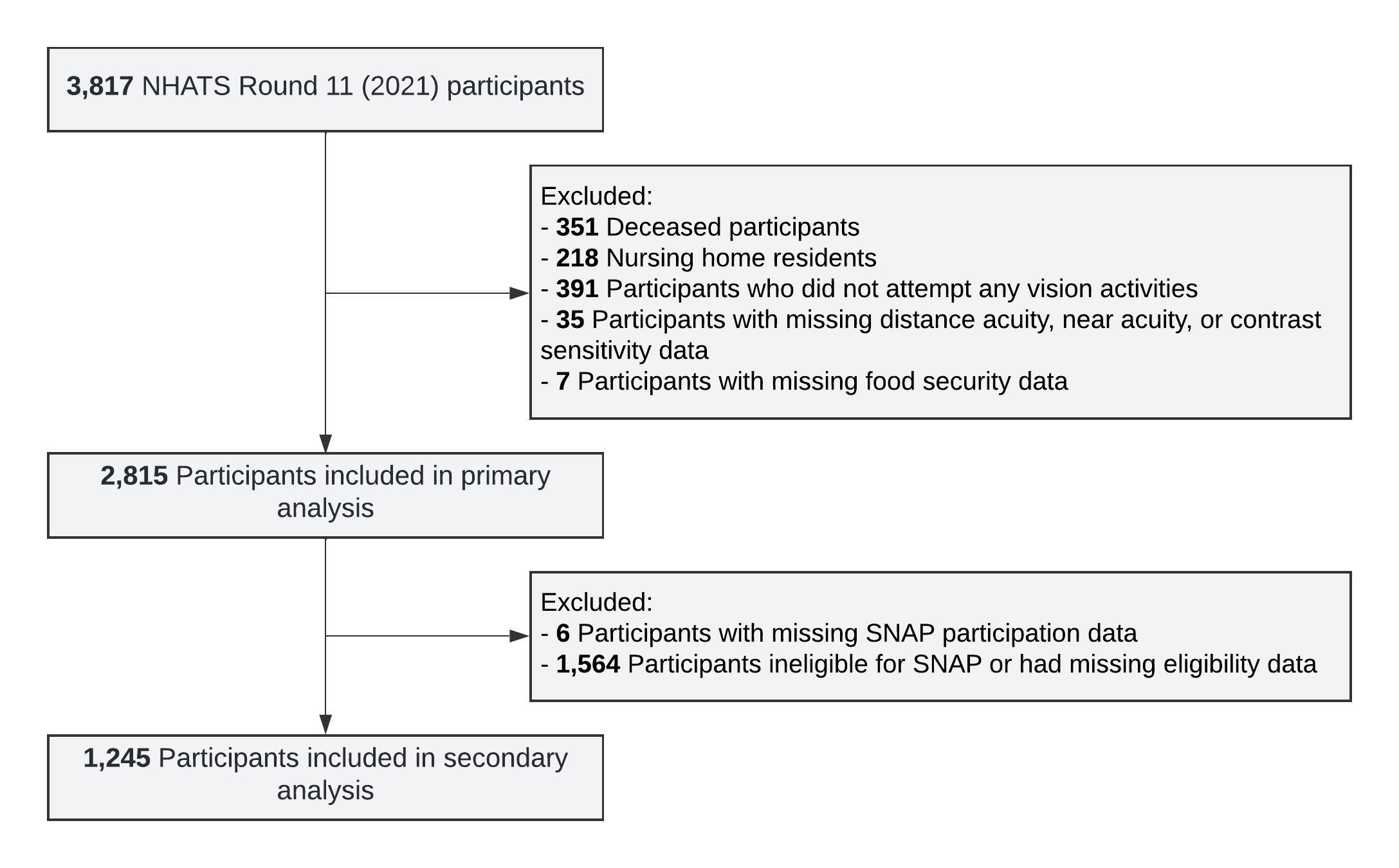


**Supplement 1**. Flow diagram of number of participants included in the study. NHATS: National Health and Aging Trends Study.
